# Supplementary material for: CFIm25 regulates human stem cell function independently of its role in mRNA alternative polyadenylation
Source: RNA Biol. 2022 May 1;19(1):686–702. doi: 10.1080/15476286.2022.2071025 (PMC9067535; doi:10.1080/15476286.2022.2071025)
Supplement: Supplemental Material [file KRNB_A_2071025_SM4450.zip › Supplemental Figure 1 to 6.pdf]

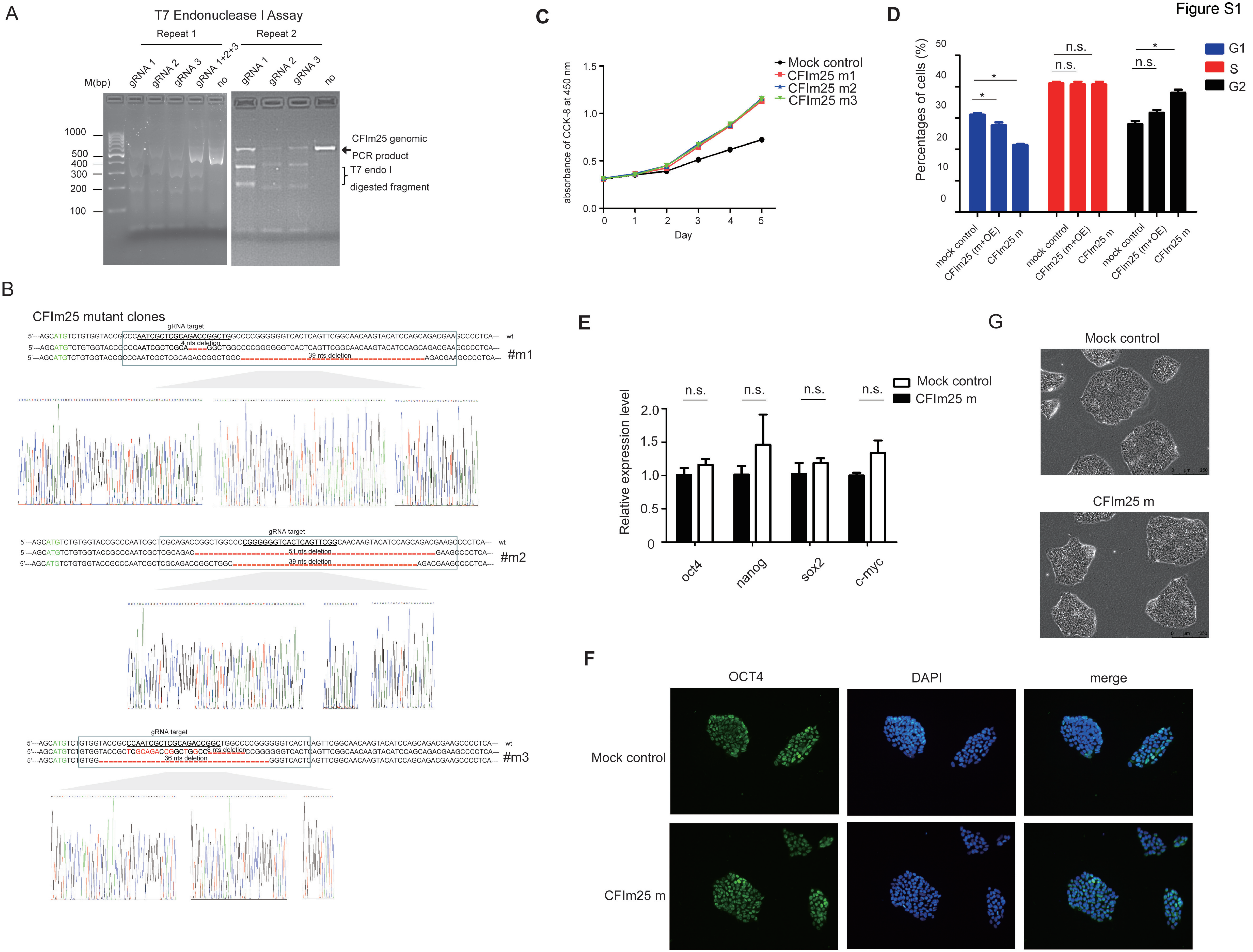

Figure S1 (A) SYBR Green staining of PCR product and DNA fragments resulted from T7 Endonuclease I assay. PCR product amplified from CFIm25 gene locus is indicated by the arrow and digested DNA fragments is indicated by the bracket. Two representative replicates experiments are shown. (B) Sanger sequencing of CFIm25 gene locus to confirm the genomic mutations/deletions in three CRISPR-Cas9 system-mediated H9 cell clones. Start codon 'ATG' is colored green, nucleotides colored in red represent mutations, and symbol "-" stands for nucleotide deletion at the corresponding position. Genomic positions targeted by gRNAs are underlined. (C) Cell proliferation rate measurement by CCK-8 kit in mock and three CFIm25-mutant H9 cell lines. The starting cell density in this experiment is 7500 cell per well of 96 well plates. Three independent experiments have been carried out and representative results are shown. (D) Quantifications of the percentages of cells at different stages during cell cycles. The results are from three independent experiments. A representative result is shown in Figure 1D (m: CFIm25-m3; OE: overexpression). Student's t-test was used to estimate the significance of the change. *P<0.05; n.s.: non-significant. (E) RT-qPCR analysis of the expression level of four pluripotency-associated markers in mock and CFIm25-m (1-3) hESCs. (F) Immunostaining analysis of pluripotency marker OCT4 in mock and CFIm25-m3 hESCs. (G) Phase-contrast images of mock and CFIm25-m3 hESC clones.

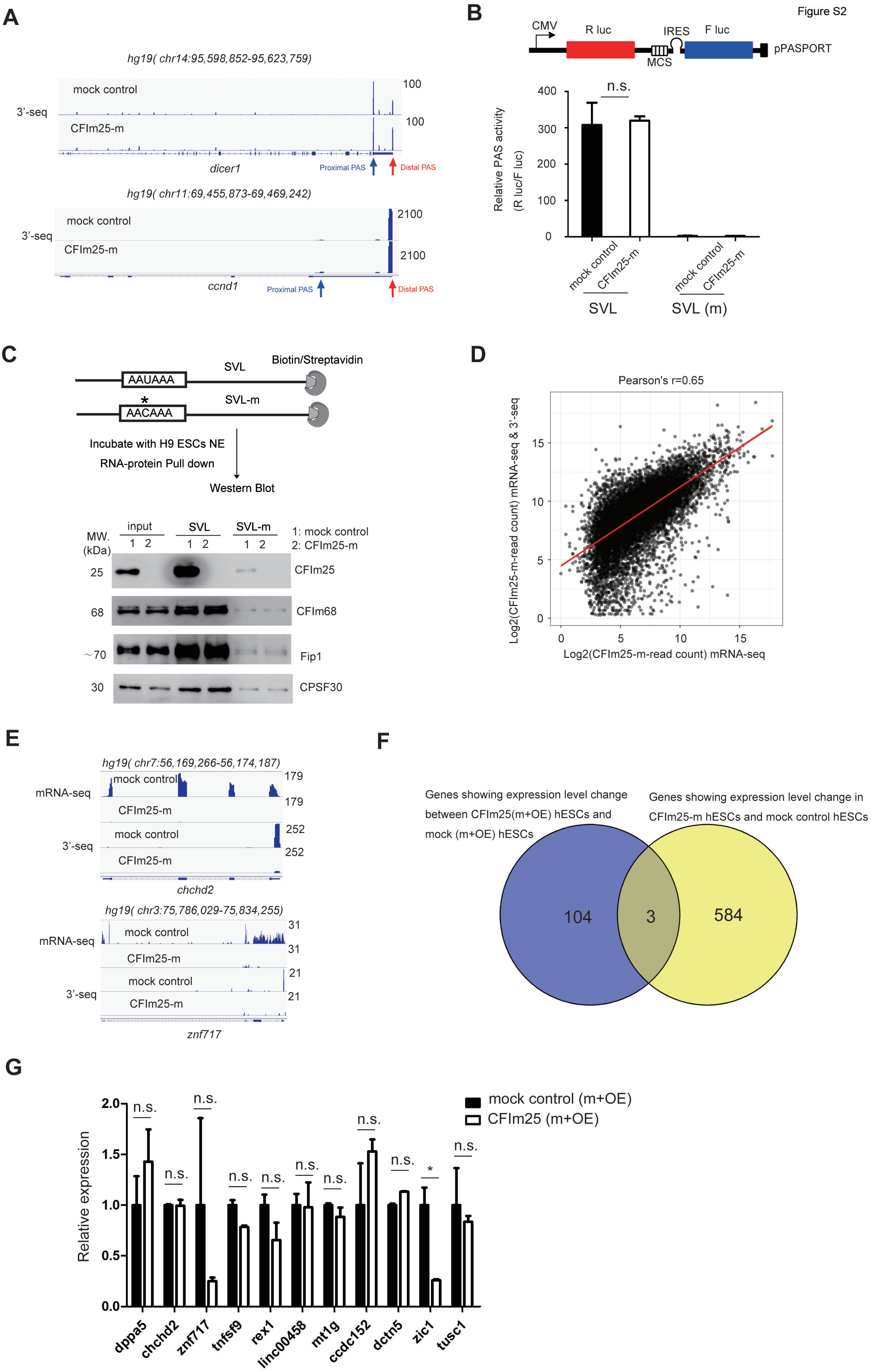

Figure S2. (A) IGV track screen shots showing the 3'-seq results for dicer1 and ccnd1 genes in mock and CFIm25-m H9 cells. Two predominant PASs within 3'UTR regions are indicated with arrows. Proximal or distal PAS are named according to their positions relative to gene 5' end. (B) Measurement of canonical SVL PAS processing efficiency using pPASPORT system in mock and CFIm25-m cells, SVL-m (AAUAAA core hexamer was replaced as AACAAA) serves as negative control. SVL or SVL(m) PAS were inserted into multiple cloning sites between Renilla luciferase (Rlu) gene and IRES (internal ribosome entry site). Downstream of the IRES is the Firefly luciferase (Flu) gene. Relative PAS processing efficiency was quantified by calculating the Rlu/Flu ratio. Results from three independent experiments are quantified and represented. Student's t-test was used to estimate the significance: ns: non-significant. (C) Schematic representation of the SVL RNA substrates used in the biotin-streptavidin pull-down assay (top). The AAUAAA hexamer in wild-type RNA substrate and AACAAA in mutant substrate (boxes) are shown. The asterisk is used to highlight the single nucleotide change. Bottom panel shows the western blot results of known core 3' processing factors in the RNA-biotin based pull-down experiment using nuclear extracts (NEs) prepared from mock and CFIm25-m H9 cells. Two independent experiments have been carried out and representative results are shown. 5% of the lysate was kept as input. The primary antibody for CFIm25 is from santa cruz (sc-81109). (D) Comparison of gene expression profiling by 3'-seq and mRNA-seq. X axis: total read count for each gene in CFIm25-m sample based on mRNA-seq data; Y axis: total read count for each gene in CFIm25-m based on mRNA-seq results of control and 3'-seq analysis. The mRNA-seq read count for a gene in CFIm25-m sample=(mRNA-seq read count for this gene in control H9) x (expression fold change for this gene based on 3'-seq analyses: CFIm25-m/control). Both X axis and Y axis are in log scale. Pearson's r=0.65. (E) IGV track screen shots showing mRNA-seq and 3'-seq results for chchd2 and znf717 genes in mock and CFIm25-m H9 cells. (F) Venn diagram showing the number of overlapping and non-overlapping genes that display expression level change upon CFIm25 (m+OE) (blue) and CFIm25-m (yellow) (m: mutant; OE: overexpression). (G) Comparison of the expression level of indicated genes in mock (m+OE) and CFIm25 (m+OE) H9 cells. Data comes from RNA-seq analysis listed in Supplemental Table 4. Student's t-test was used to estimate the significance: *p<0.05; ns: non-significant.

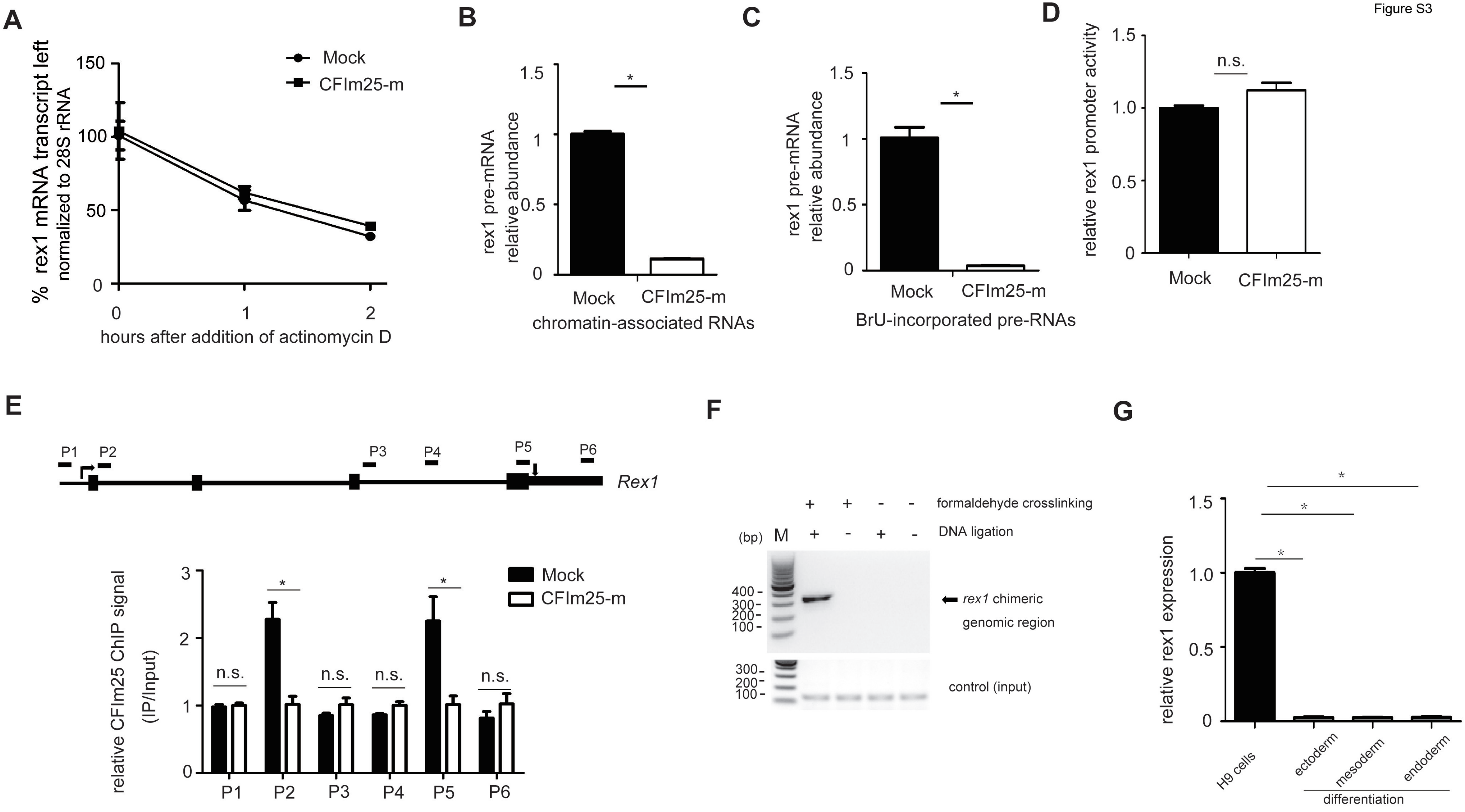

Figure S3 (A) Mock and CFIm25-m (1-3) H9 cells were subjected to Actinomycin D treatment and total RNAs were extracted at the indicated time. RT-qPCR was used to calculate the percentage of the rex1 mRNA left, 28s rRNA serves as normalization control. (B-C) RT-qPCR analysis of rex1 pre-mRNA abundance using chromatin-associated RNAs (B) and BrU-incorporated pre-mRNAs (C) in mock and CFIm25-m H9 cells. gapdh gene product serves as internal normalization control. (D) Comparison of rex1 gene promoter activity in control and CFIm25-m H9 cells using pGL3-basic reporter system. Student's t-test was used to estimate the significance of the change. ns: non-significant. (E) ChIP-qPCR analysis using primary antibody against CFIm25 and indicated primers targeting different position of rex1 gene locus. For the bar graph, y axis represents the fold change of ChIP signal in mock H9 cells in comparison to that of CFIm25-m cells. x axis stands for the indicated positions across rex1 gene locus. (F) PCR product resulting from 3C library preparation in the presence/absence of formaldehyde crosslinking and DNA ligation. (G) RT-qPCR analysis of rex1 gene expression in undifferentiated H9 cells, and trilineage differentiated cells. Gapdh mRNA was assayed as normalization control. Student's t-test was used to estimate the significance of the change. *P<0.05.

Figure S4

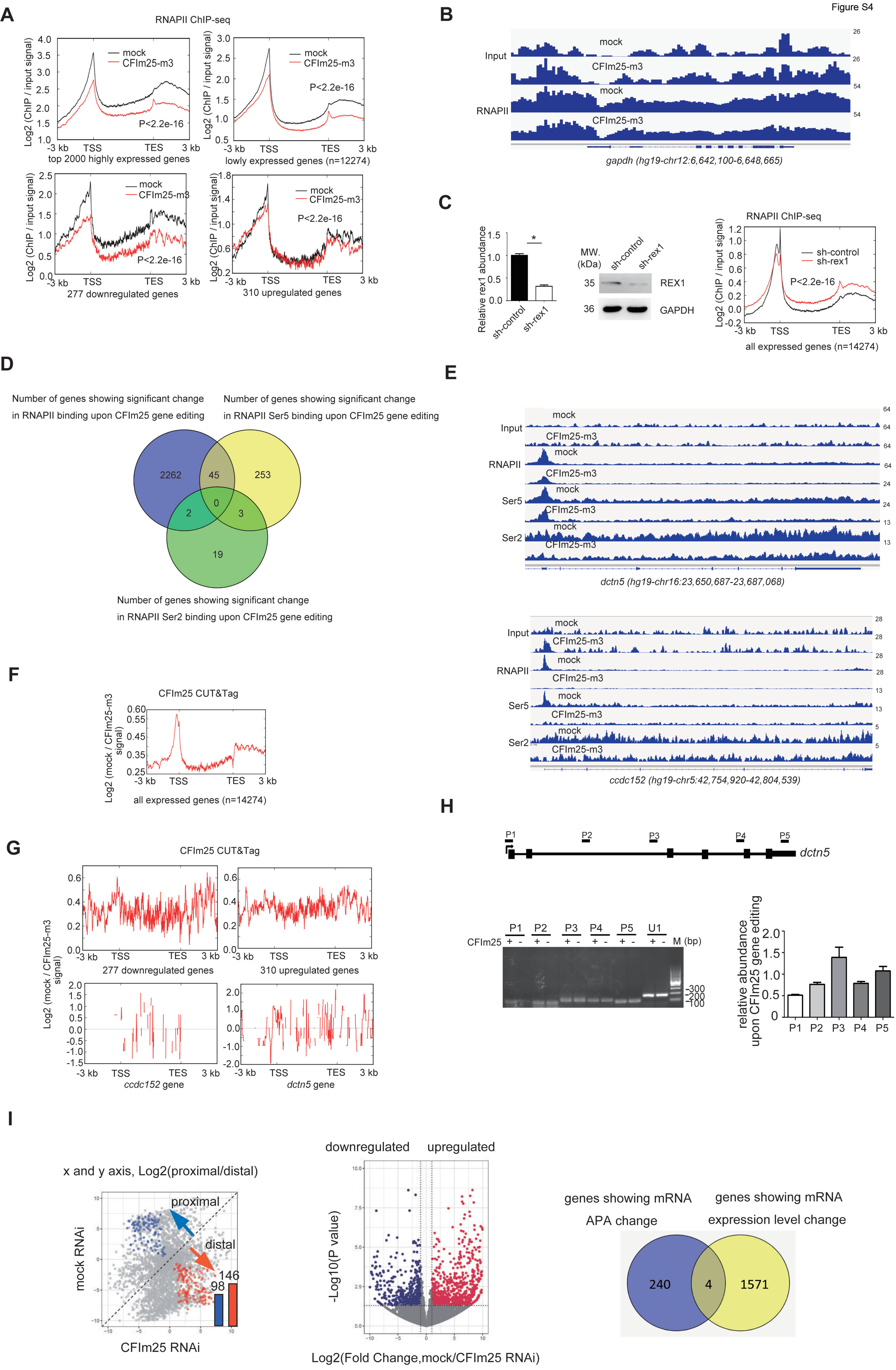

Figure S4. (A) Metagene plots of RNAPII ChIP-seq reads for highly expressed genes (top 2000 genes based on mRNA-seq FPKM value), lowly expressed gene (the rest of the genes), 277 down-regulated genes and 310 up-regulated genes upon CFIm25 gene editing. K-S test was used to examine the significance of the difference between the two plots. (B) IGV track screen shot showing RNAPII ChIP-seq result for gapdh gene in mock and CFIm25-m H9 cells. (C) Metagene plots of RNAPII ChIP-seq reads for actively expressed genes in mock and rex1 RNAi H9 cells. K-S test was used to examine the significance of the difference between the two plots. The rex1 gene knockdown efficiency was estimated by RT-qPCR and western blot analysis. Student's t-test was used to estimate the significance of the change. *P<0.05. (D) Venn diagram showing the number of genes that displayed differential RNAPII/Ser5/Ser2 binding upon CFIm25 depletion. (E) IGV track screen shots showing RNAPII, RNAPII Ser5 and Ser2 ChIP-seq results for dctn5/ccdc152 gene in mock and CFIm25-m H9 cells. (F) Metagene plots of CFIm25 CUT-Tag reads for actively expressed genes in mock and CFIm25-m H9 cells. CFIm25 CUT-Taq profile in CFIm25-m cells was used as normalization control. (G) Plots showing the normalized CFIm25 CUT&Tag signals in the specific group of genes (up-regulated or down-regulated genes) or individual gene (ccdc152, dctn5). (H) Nuclear run-on assay on the nascent dctn5 transcript. The gene structure and the probe positions are indicated on the top. The diagram for the nuclear run-on assay is shown in the Figure 4E. A representative set of RT-PCR data are shown in the middle panel. RT-PCR products from mock and CFIm25-m H9 cells are indicated below each set. 'CFIm25 -' represents CFIm25-m cell nuclei, whereas 'CFIm25 +' represents mock cell nuclei. Bar graph represents RT-qPCR data from three independent experiments. U1 snRNA was assayed as normalization control. Student's t-test was used to estimate the significance of the change. *P<0.05. (I) Comparison of global mRNA APA (left) and gene expression profiles (middle) in control and CFIm25 RNAi cells using the previously reported dataset. The plots are similar to that in Figure 2A-B. Venn diagram shows that the two groups of genes showing changes do not overlap extensively.

Figure S5

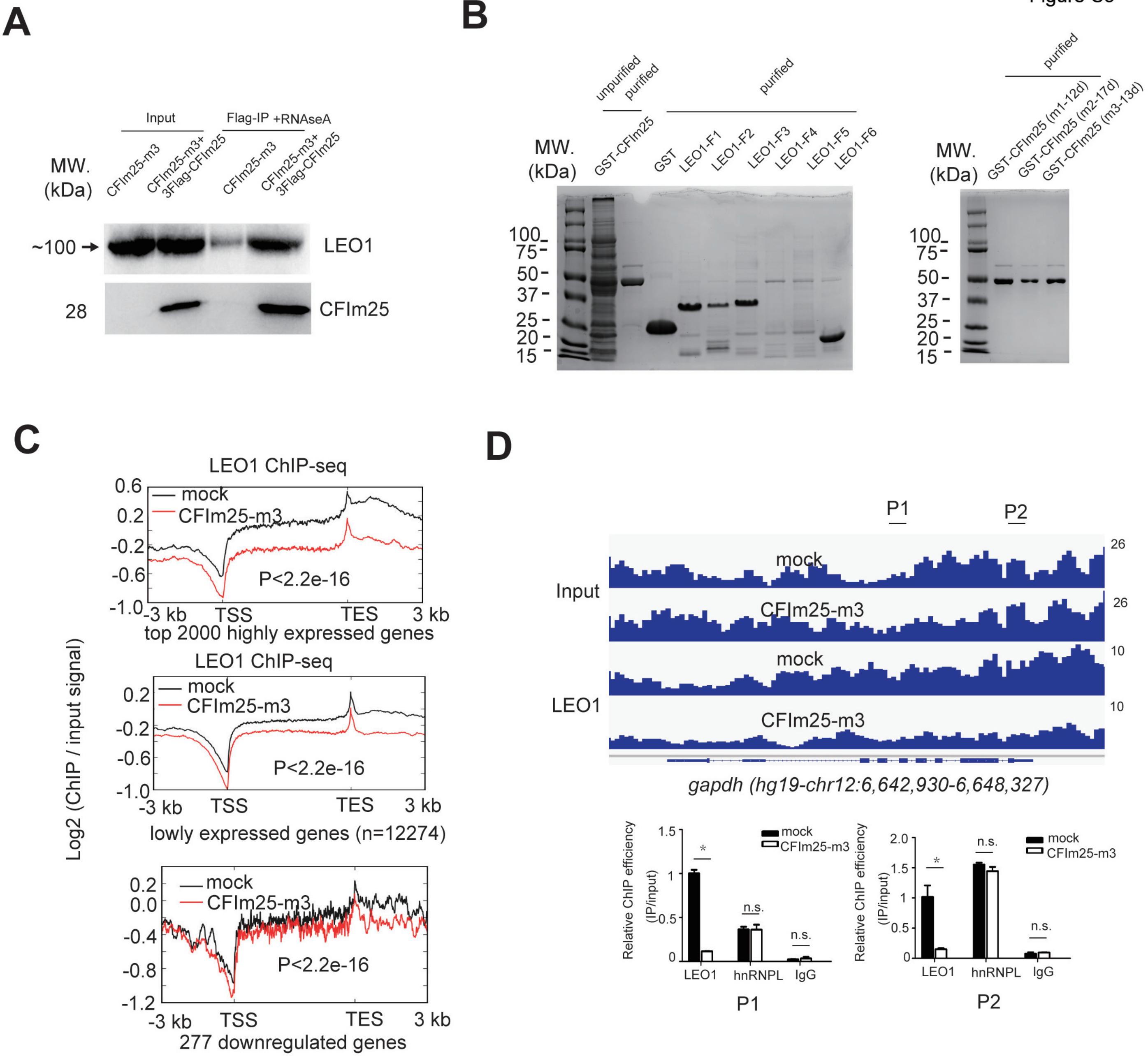

Figure S5.(A) Western blotting analysis of the abundance of LEO1 protein in Flag-IPed sample. Flag-IP was performed using extracts from CFIm25-m3 and CFIm25(m3+3XFlag-CFIm25 overexpression) H9 cells in the presence of 5 ug/ml RNAseA. Input:1% of the lysates for IP. (B) Coommassie blue staining of purified GST-CFIm25, His-LEO1 (truncation fragments 1-6) fusion proteins (left), and three GST-CFIm25 mutants (the N terminus mutations are based on sequences listed in Figure S1B. (C) Metagene plots of LEO1 ChIP-seq reads for highly expressed genes (top 2000 genes based on mRNA-seq FPKM value) (top), lowly expressed gene (the rest of the genes). K-S test was used to examine the significance of the difference between the two plots. (D) IGV track screen shot showing LEO1 ChIP-seq result for gapdh gene in mock and CFIm25-m H9 cells. Below is the ChIP-qPCR data amplifying two indicated genomic regions of gapdh gene. Student's t-test was used to estimate the significance of the change. *P<0.05. n.s.:non-significant.

Figure S6

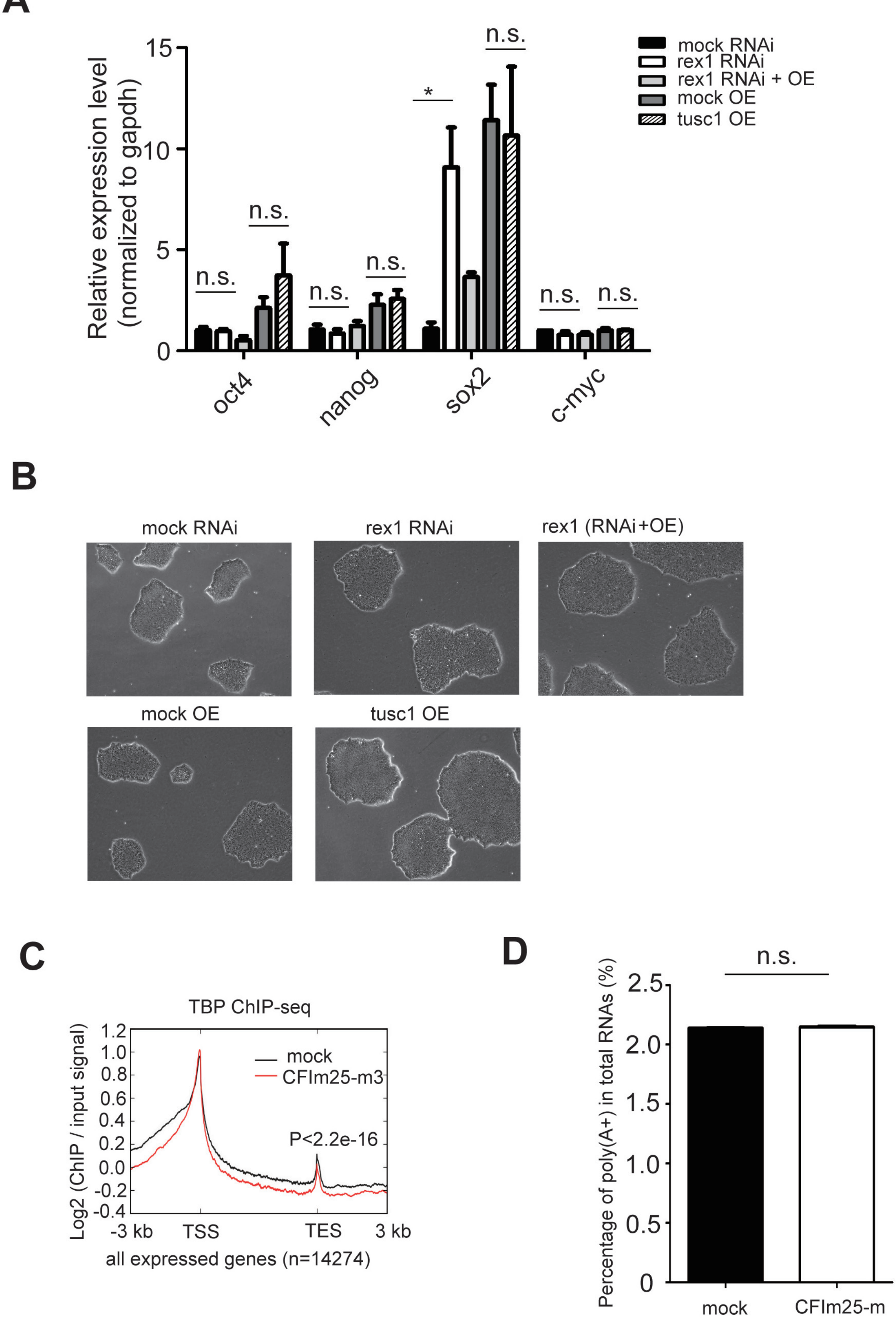

Figure S6. (A) RT-qPCR analysis of the expression level of four pluripotency-associated markers in indicated cell lines. Student's t-test was used to estimate the significance of the change. *P<0.05; ns: non-significant. (B) Representative phase-contrast images of indicated cell lines. (C) Meta-gene plots of TBP ChIP-seq reads for all expressed genes in control and CFIm25-m3 cells. (D) Bar graph showing the percentages of poly(A+) RNAs among total RNAs in mock and CFIm25-m H9 cells. Poly (A+) RNAs were purified by OligodT magnetic beads from total RNAs. Quantification was performed with three independent experiments. Student's t-test was used to estimate the significance of the change. ns: non-significant.
