# Supplementary material for: CFIm25 regulates human stem cell function independently of its role in mRNA alternative polyadenylation
Source: RNA Biol. 2022 May 1;19(1):686–702. doi: 10.1080/15476286.2022.2071025 (PMC9067535; doi:10.1080/15476286.2022.2071025)
Supplement: Supplemental Material [file KRNB_A_2071025_SM4450.zip › video_files.docx]

supplementary video 1 link:

<https://data.cyverse.org/dav-anon/iplant/home/ranyy/Supplementary%20Video%201%20control%20hESC%20induction%20to%20cardiomyocytes%20.wmv>

supplementary video 2 link:

<https://data.cyverse.org/dav-anon/iplant/home/ranyy/Supplementary%20Video%202%20CFIm25%20mutant%20hESC%20induction%20to%20cardiomyocytes%20.wmv>
